# The “Great Debate” at Immunotherapy Bridge 2021, December 1st–2nd, 2021

**DOI:** 10.1186/s12967-022-03384-w

**Published:** 2022-04-21

**Authors:** Paolo A. Ascierto, Lisa H. Butterfield, Olivera J. Finn, Andrew Futreal, Omid Hamid, Theresa LaVallee, Michael A. Postow, Igor Puzanov, Jeffrey Sosman, Bernard A. Fox, Patrick Hwu

**Affiliations:** 1 Department of Melanoma, Cancer Immunotherapy and Innovative Therapy, Istituto Nazionale Tumori IRCCS “Fondazione G. Pascale”, Naples, Italy; 2 Microbiology and Immunology, Parker Institute for Cancer Immunotherapy, University of California, San Francisco, CA USA; 3 Department of Immunology, University of Pittsburgh School of Medicine, Pittsburgh, PA USA; 4 Division of Cancer Medicine, Department of Genomic Medicine, The University of Texas MD Anderson Cancer Center, Houston, TX USA; 5 Medical Oncology, The Angeles Clinic and Research Institute, A Cedar-Sinai Affiliate, Los Angeles, CA USA; 6 Parker Institute for Cancer Immunotherapy, San Francisco, CA USA; 7 Memorial Sloan Kettering Cancer Center and Weill Cornell Medical College, New York, NY USA; 8 Department of Medicine, Roswell Park Comprehensive Cancer Center, Buffalo, NY USA; 9 Division of Hematology/Oncology, Department of Medicine, Northwestern University Feinberg School of Medicine, Chicago, IL USA; 10 Earle A. Chiles Research Institute, Robert W. Franz Cancer Research Center, Providence Cancer Institute, Portland, OR USA; 11 Moffitt Cancer Center, Tampa, FL USA

**Keywords:** Immunotherapy, Cancer vaccine, Checkpoint inhibitors, Nivolumab, Pembrolizumab, Overall survival, Progression-free survival, Clinical trials

## Abstract

As part of the 2021 Immunotherapy Bridge virtual congress (December 1–2, Naples, Italy), the Great Debate sessions featured experts who were assigned counter opposing views on four important questions in immunotherapy today. The first topic was whether oncolytic viruses or other specific immunomodulators were the more promising approach for intralesional therapy. The second was whether early surrogate endpoints, such as response rate or progression-free survival, correlate with long-term overall survival was considered. Thirdly, whether vaccines can transform cold into hot tumors was discussed and, finally, broad versus deep analytic profiling approaches to gain insights into immune-oncology development were compared. As with previous Bridge congresses, presenters were invited by the meeting Chairs and positions taken during the debates may not have reflected their respective personal view. In addition, the views summarised in this article are based on available evidence but may reflect personal interpretation of these data, clinical experience and subjective opinion of the speaker.

## Introduction

As part of the 2021 Immunotherapy Bridge virtual congress (December 1–2, Naples, Italy), the Great Debate session featured counterpoint views from leading experts on four clinical questions in immunotherapy today. The first topic was whether oncolytic viruses or other specific immunomodulators were the more promising approach for intralesional therapy. The second was whether early surrogate endpoints, such as response rate or progression-free survival, correlate with long-term overall survival was considered. Thirdly, whether vaccines can transform cold into hot tumors was discussed and, finally, broad versus deep analytic profiling approaches to gain insights into immune-oncology development were compared.

For each of the selected topics, two experts presented the argument and counterargument in support of two different points of opinion. As with previous Bridge congresses, the debates were assigned by meeting Chairs and the positions held by the respective experts during the debates may not have necessarily reflected their own personal opinions. Discussions summarised in this article are evidence-based but may reflect clinical experience, personal interpretation, and subjective view of the speaker. These discussions are not intended as a rigorous evaluation of the respective subject but instead reflect two opposing interpretations in order to allow the consideration of different views. The virtual audience were asked to vote on which view they favored both before and after the debate. Discussion of these important topics are summarised in this report.

## Intralesional therapy: oncolytic viruses versus other specific immune modulators

### Igor Puzanov: in favor of oncolytic viruses

Tumor-directed immunotherapy involves focusing immune activation on the most relevant part of the immune system with the aim of improving antitumor activity as well as reducing immune-related adverse events. An immune-active TME type I interferon (IFN) transcriptional signature has been shown to be associated with greater clinical benefit from checkpoint immune blockade. Intratumoral immunotherapies include specific immunomodulators, such as toll‐like receptor (TLR)-9 agonists as well as oncolytic viral therapies, such as talimogene laherparepvec (T‐VEC). However, which locoregional approach is better at sensitizing tumors to immune checkpoint inhibitor therapy and promoting an abscopal effect remains unclear.

TLR agonists can induce a local IFN-α gene signature, upregulate antigen-presenting cells and increase lymphocyte infiltration into tumors. However, they do not promote antigen release other than by destruction of the tumor by injection. The TLR-9 agonist tilsotolimod was investigated in combination with ipilimumab or pembrolizumab in a phase I/II study in patients with metastatic melanoma. In 49 evaluable PD-1-refractory patients, ORR was 22.4% and the median duration of response was 11.4 months. The disease control rate (DCR) was 71.4%. Overall, 48% of patients had ≥ grade 3 toxicities [1]. However, in the phase II ILLUMINATE-301 trial, the combination of tilsotolimod plus ipilimumab failed to meet the primary endpoint of improved ORR versus ipilimumab alone (8.8% vs 8.6%) in patients with advanced melanoma who are refractory to a PD-1 inhibitor [2].

Tumor infection with oncolytic viruses results in type I IFN production and immunogenic cell death. Unlike TLR agonists, oncolytic viruses can induce pathogen-associated molecular pattern molecules (PAMPs) as well as damage-associated molecular patterns (DAMPs). T‐VEC is an HSV‐1-derived oncolytic virus that selectively replicate in tumor cells and produces GM‐CSF to trigger the release and presentation of tumor‐derived antigens and induces a systemic antitumor immune response. Clinical trials of T-VEC combined with immune checkpoint blockade have reported both positive and negative results in melanoma. In a phase Ib trial in 198 patients with previously treated unresectable melanoma, T-VEC in combination with ipilimumab resulted in a significantly higher ORR than ipilimumab alone (39% vs. 18%; p = 0.002), with majority of these responses durable, thereby meeting the primary endpoint [3].

The combination also had a manageable safety profile. However, in the phase III MASTERKEY-265 study in 692 patients with advanced melanoma, T-VEC in combination with pembrolizumab failed to meet its PFS primary endpoint. At a median follow-up of 31.0 months, there was a 5.8-month difference in median PFS between T-VEC plus pembrolizumab versus pembrolizumab alone, but this was not significant (14.3 months versus 8.5 months; HR 0.86, p = 0.13) [4]. Although mature OS data are not yet reported, a survival benefit was not reported at the interim analysis (median not yet reached with T-VEC plus pembrolizumab compared with 49.2 months with pembrolizumab alone; HR 0.96; p = 0.74) and the likelihood of achieving a significant OS benefit did not cross futility threshold.

Reasons for the lack of benefit observed with T-VEC and pembrolizumab in this trial and possible pitfalls with the wider use of oncolytic viruses, may include patient selection and the lack of easily obtainable biomarkers. Even though baseline patient characteristics were balanced between treatment arms, it is possible that patient selection led to inadvertent selection of patients who may have derived benefit from pembrolizumab alone, given that around two-thirds were PD-L1 positive and that majority had baseline lactate dehydrogenase levels within normal limits. The injection technique required for intratumoral injections and the need for the appropriate volume may also have been an issue. Subgroup analyses by region showed a significant benefit for T-VEC in the USA, which may reflect more experience with oncolytic virus administration in this country. The choice of combination selection, i.e., anti-CTLA-4 or anti-PD-1 inhibitor, and the choice of virus backbone and additional payload may also influence the efficacy of oncolytic virus therapy.

In conclusion, tumor-directed oncolytic viruses preferentially replicate in cancer cells to promote immunogenic cell death and may provide the optimum means to generate patient-specific antitumor immune responses. They lead to the induction of localized inflammation, release of both PAMPs and DAMPs, activating both innate and adaptive immunity. They systemically activate the immune system against the tumor antigens released and can be ‘armed’ with additional genes to augment the natural properties of the virus with additional mechanisms of action. There are multiple oncolytic trials ongoing, varying viral backbone, delivery mode, additional payloads and combination strategies. It remains to be seen whether any of these strategies will lead to superior outcomes.

### Omid Hamid: in favor of immune modulators

There is a boom in intratumoral therapy utilizing several solid strategies with differing mechanisms of action in clinical development. These strategies have produced significant initial results with evidence of immune activation. In addition to oncolytic viruses, newer therapeutics include various immune agonists, in particular TLR agonists, retinoic-inducible gene-I (RIG-I)-like receptor agonists, and stimulator of IFN-induced gene (STING) agonists. The future is promising with these agents and their second-generation iterations.

In relation to oncolytic viruses, T-VEC with pembrolizumab had no significant beneficial effect compared with pembrolizumab alone in a phase III trial [4]. Interim analysis of OS indicated no benefit with the combination. Studies with other oncolytic viruses in other cancers, e.g., pexastimogene devacirepvec (Pexa-Vec) combined with sorafenib in liver cancer, have also failed to show any benefit, suggesting a pattern of multitumor failure. The use of oncolytic viruses has multiple challenges, including penetration into the tumor, antiviral immune responses, off-target infection, hypoxia in the TME, and a lack of predictive biomarkers. In addition, oncolytic viruses come at considerable cost; the cost of adding T-VEC to ipilimumab was approximately $1.6 million to gain one additional progression-free quality-adjusted life-year, one additional progression-free life-year, or to have one additional patient attain an objective response [5]. Many practitioners have failed to accept this therapy even in the approved population due to obstacles in pharmacy approval and preparation.

TLR-9 agonists augment function of antigen-specific CD8+ T cells and, in melanoma, have shown evidence of immunogenicity in combination with cancer vaccines and promising activity as a single-agent or in combination with anti-PD-1 blockade in PD-1 naïve and PD-1-refractory patients. In a phase Ib trial, the type A CpG TLR-9 agonist vidutolimod (CMP‐001) plus pembrolizumab resulted in durable responses in 25% in patients with melanoma refractory to PD‐1 inhibition [6]. Patients who responded had non-inflamed tumors at baseline and induction of an IFNγ gene signature following treatment. In another trial, neoadjuvant CMP-001 in combination with nivolumab was a viable approach, with acceptable toxicity and promising efficacy, indicated by a major pathological response rate of 60%, in patients with regionally advanced melanoma [7]. Patients who responded had evidence of activated peripheral CD8+ T cells, with increased expression of CD25, granzyme B and perforin, while non-responders had increased expression of CD27 and lymphocyte-activation gene (LAG)-3. In human melanoma cell lines resistant to anti-PD-1 therapy due to JAK1/2 knock-out mutations, administration of the intratumoral TLR-9 agonist SD101 with anti-PD-1 overcame this resistance by the activation of IFN signalling and the stimulation of natural killer (NK) cells [8].

Intratumoral administration of TLR agonists can lead to rapid efflux from the tumor, resulting in acute systemic drug exposure and transient but high level of peripheral proinflammatory cytokines. TransCon TLR-7/8 agonist was developed to provide sustained local release of resiquimod following administration of a hydrogel depot. In a syngeneic murine tumor model, TransCon TLR-7/8 agonist elicited sustained expression of cytokines and inflammatory chemokines in the tumor but with low levels in plasma, promoted sustained expression of peripheral B, T and KN cells, and was associated with a potent antitumor response [9].

Another approach has been to conjugate a polyspecific integrin-binding peptide to a CpG TLR-9 agonist to generate a tumor-targeted immunomodulatory agent (PIP-CpG) that can be delivered by intravenous infusion. Systemic delivery of PIP-CpG induces tumor regression and enhances therapeutic efficacy compared with untargeted CpG in murine breast and pancreatic cancer models [10]. PIP-CpG also transforms an immunosuppressive TME in which myeloid-derived suppressor cells are predominant into a lymphocyte-rich TME infiltrated with activated CD8+ T cells, CD4+ T cells, and B cells.

Intratumoral anti-CTLA-4 delivery offers the potential for increased efficacy but with lower toxicity than intravenous administration [11]. Intratumoral checkpoint blockade combined with other intratumoral therapy may create an in situ vaccination effect with systemic benefit. BO-112, a nanoplexed form of polyICLC, can restore sensitivity to PD-1 therapy in refractory patients and, in combination with pembrolizumab for patients with unresectable stage III or IV melanoma with confirmed progression on PD-1/PD-L1 inhibitors, demonstrated an ORR of 27% among 37 evaluable patients [12]. The LUD2014-011 trial was initiated to test the safety and clinical activity of intratumoral CTLA-4 blockade with tremelimumab plus the intratumoral TLR3 agonist polyICLC plus systemic PD-L1 blockade with durvalumab in patients with advanced solid tumors. In 17 patients with treatment-refractory recurrent breast cancer, the combination was safe and produced clinical responses in patients with advanced triple-negative breast cancer (TNBC) and non-TNBC [13]. Treatment was associated with enhanced intratumoral immune effectors and markers of T cell function, with increased CD8+ T cells expressing LAG-3 and T cell immunoglobulin and mucin domain-containing protein (TIM)-3, PD-L1+ tumor cells and stromal cells.

Intratumoral ipilimumab has also been assessed in combination with systemic (intravenous [IV]) administration of nivolumab. In the NIVIPIT trial in 61 patients with previously untreated metastatic melanoma, intratumoral ipilimuamab in combination with IV nivolumab resulted in lower toxicity than IV ipilimumab with IV nivolumab [14]. ORR was 50% in the intratumoral ipilimumab group versus 65% in the IV ipilimumab group. Intratumoral administration of the triple combination of low-dose anti-CD137, anti-OX40, and anti-CTLA-4 10 has also been shown to be more effective than systemic administration [15].

Electroporated plasmid interleukin-12 (tavokinogene telseplasmid) is a novel pro-inflammatory intratumoral therapy with substantial single agent activity in melanoma. Interim data from the KEYNOTE-695 trial of patients with stage III/IV melanoma actively progressing on anti-PD-1 therapy has reported durable responses in locally treated and distant visceral metastatic untreated lesions with limited systemic toxicity [16].

**Fig. 1:**
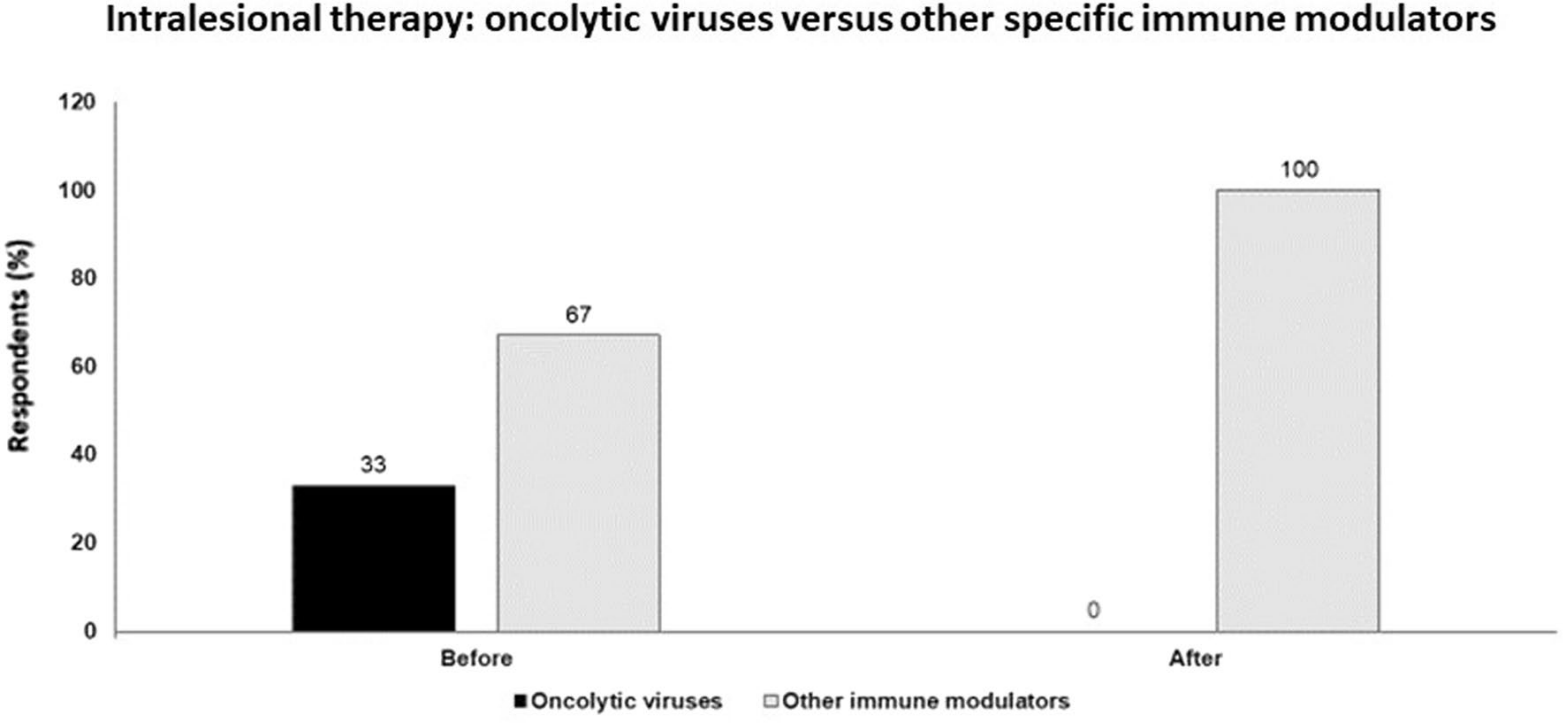
Intralesional therapy: oncolytic viruses versus other specific immune modulators; audience response before and after debate

Intratumoral immunotherapies have the capability to trigger a systemic antitumor immune response across multiple tumor types. Combination with immune checkpoint inhibitors is the predominant focus in the development of these therapies. Such combinations may improve the depth of response and may benefit patients other than just those who have relapsed or are refractory to checkpoint blockade. Sequencing of treatments for optimal benefit is important, as is correlative analysis of tumor/blood for precision-based approaches (Fig. 1).

### Key points

- Tumor-directed oncolytic viruses preferentially replicate in cancer cells to promote immunogenic cell death and may provide the optimum means to generate patient-specific antitumor immune responses.
- Oncolytic virsuses are highly inflammatory, systemically activate the immune system against the tumor antigens released, and can be ‘armed’ with additional genes to augment the natural properties of the virus.
- However, oncolytic viruses have multiple challenges, including penetration into the tumor, antiviral immune responses, off-target infection, hypoxia in the TME, a lack of predictive biomarkers, and high costs.
- Intratumoral immune modulators, such as TLR agonists, have the capability to trigger a systemic antitumor immune response across multiple tumor types.
- TLR agonists have shown promising activity as a single-agent or in combination with anti-PD-1 blockade and may improve depth of response and benefit patients other than just those who have relapsed or are refractory to checkpoint blockade.

## Do early surrogates correlate with long-term overall survival? Yes or no

### Michael A. Postow: yes

Early surrogates for long-term OS in clinical trials generally refer to response rates and progression free survival (PFS). For each of the main systemic treatment modalities for melanoma (anti-PD-1 therapy, combined PD-1 and CTLA-4 blockade, and targeted BRAF and MEK inhibition), response rates have been shown to correlate with OS; PFS also has been shown to correlate with OS.

In a pooled analysis of the KEYNOTE-001 and -006 trials of PD-1 monotherapy with pembrolizumab in melanoma, 4-year OS rates were 95.2%, 73.0% and 47.7%, for patients with complete response, partial response or stable disease respectively, at week 12 [17]. Thus, clearly response at week 12 is associated with longer-term OS. This trial used the Response Evaluation Criteria in Solid Tumors (RECIST) response, which may not capture the full benefit of immunotherapy. However, analysis of the KEYNOTE-001 trial using both RECIST and immune-related response criteria (irRC) showed that patients without progression using both criteria had the best OS, whereas those with progressive disease by both criteria had the worst OS [18]. Patients with progressive disease per RECIST but not irRC also had worse OS than patients without progression by RECIST and irRC. Thus, OS is worse based upon response and progression assessments irrespective of whether standard RECIST or irRC criteria is used.

Results are similar with combined PD-1 and CTLA-4 blockade. In the CheckMate-067 study of nivolumab plus ipilimumab versus nivolumab or ipilimumab alone, patients with best ORR of complete or partial responses had better OS at 5 years across all three treatment arms [19], showing that early surrogates do correlate with longer-term OS. In the Adaptively Dosed ImmunoTherapy Trial (ADAPT-IT), patients received two doses of nivolumab plus ipilimumab followed by a computed tomography scan at week 6. Patients without new lesions or index lesion tumor growth of > 4% were defined as having early favorable antitumor effect (FATE) and switched to nivolumab monotherapy whereas patients without FATE at week 6 received third and fourth doses of the combination followed by nivolumab monotherapy [20]. In 41 patients who had FATE at week 6, response rates (best ORR by RECIST 58%) and toxicity were similar to those seen with four doses. No patients with more than one growing tumor at week 6, so at a very early timepoint, had a later RECIST response; FATE was associated with favorable OS (80% at a median follow-up of 25 months).

Similarly, OS is associated with best ORR to targeted therapy. Pooled analyses of the COMBI-d and COMBI-v trials of the BRAF inhibitor dabrafenib plus MEK inhibitor trametinib in patients with unresectable or metastatic melanoma showed that complete response was associated with improved 5-year OS rate [21]. PFS is also associated with OS. A systematic review of 60 checkpoint inhibitor trials reported that 6-month PFS was strongly correlated with and could be used to predict 12-month OS [22].

In conclusion, treatment response does appear to be a good predictor of longer-term OS and, while imperfect, PFS is also predictive of OS with immune checkpoint blockade.

### Jeffrey Sosman: no

In answer to a slightly different question, do early biologic surrogates correlate with OS, the answer is no. Both clinical surrogates, such as tumor response and PFS, as well as some biomarkers, do correlate with OS. However, the more important question I will focus on is instead whether early surrogates can assist in selection of therapy, the answer to which is currently no.

Surrogate tissue biomarkers can be measured at baseline or after therapy, assessing malignant cells or the TME, and are performed using both tumor tissue or peripheral blood (cells or serum/plasma). Biomarkers assessing malignant cells include the tumor mutational burden (TMB), specific gene alterations of cancer genes (oncogenes and tumor suppressor genes), and PD-L1 expression on malignant cells or on mononuclear cells in stroma. On the other hand, assays of the TME include a T cell inflamed gene signature as assessed by gene expression profiling and the TME architecture as assessed by multiplex immunohistochemistry (IHC).

Cancers have a wide spectrum in their number of mutations, with melanoma having one of the highest mutational loads and associated with the most benefit from checkpoint immunotherapy. However, a fraction of tumors with a T cell-inflamed gene signature do not correlate with mutational load. For example, kidney cancer has a relatively low mutational burden but an inflammatory gene expression signature. In an analysis of > 300 patient samples across various cancers, TMB and a T cell-inflamed gene expression profile had joint predictive utility in identifying responders to pembrolizumab, with most responders having both [23]. Multiplex immunofluorescence or IHC can also be used to detect immunological components of the TME that may be linked to response, e.g., density of CD8+ T cells.

In addition to TMB, the quality of the mutation is important in influencing response to immune checkpoint inhibition. Whole-exome sequencing of 249 tumors and matched normal tissue across multiple cancer types identified several genomic correlates of immune checkpoint blockade response beyond mutational burden, including somatic events in individual driver genes, global mutational signatures, and specific human leukocyte antigen-restricted neoantigens [24]. These features were often interrelated, highlighting the complexity of identifying genetic driver events that generate an immunoresponsive TME.

Using a combination of predictors, including inflamed TME (inflammatory gene expression profile) and mutation rate, might be important in helping identify responders to anti-PD-1 therapy. However, the most important question is how we can best integrate this multivariable data in a statistically and biologically meaningful way to be able to enable us to modulate these factors in non-responding patients to improve outcomes.

Biomarkers may have a role in choice of therapy in melanoma, including the choice of ipilimumab in combination with nivolumab versus nivolumab alone. Although the combination may be generally considered more effective, it would be beneficial to be able to identify those patients who do not receive additional benefit from the combination to avoid the extra toxicity. Moreover, with the combination, some patients may benefit more from either high-dose ipilimumab (3 mg/kg) with nivolumab 1 m g/kg or low-dose ipilimumab (1 mg/kg) plus nivolumab 3 mg/kg. Choice of combination or monotherapy after progression on anti-PD-1 therapy is also important.

In CheckMate-067, both the combination of ipilimumab plus nivolumab and nivolumab monotherapy have better PFS and OS at 6.5 years follow-up than ipilimumab alone, but the benefit of the combination arm over the nivolumab arm is of less magnitude and there is significant additional toxicity [25]. The only biomarker to date that looks useful is BRAF mutation status, with the combination having a PFS and OS benefit over nivolumab monotherapy in patients with BRAF-mutant melanoma but not BRAF wild-type. In the CheckMate-511 trial, both ipilimumab 3 mg/kg plus nivolumab 1 mg/kg and ipilimumab 1 mg/kg plus nivolumab 3 mg/kg had similar PFS and OS [26]. However, low-dose ipilimumab had an improved safety profile with less grade 3/4 toxicity. In this trial, responses were similar between arms across patient subgroups, including by BRAF mutation and PD-L1 status. In a retrospective study of 355 patients with metastatic melanoma resistant to anti-PD-1/PD-L1 therapy, ipilimumab plus anti-PD-1 had a higher ORR and OS than ipilimumab monotherapy [27]. However, BRAF-mutant status did not appear to favour the combination.

**Fig. 2:**
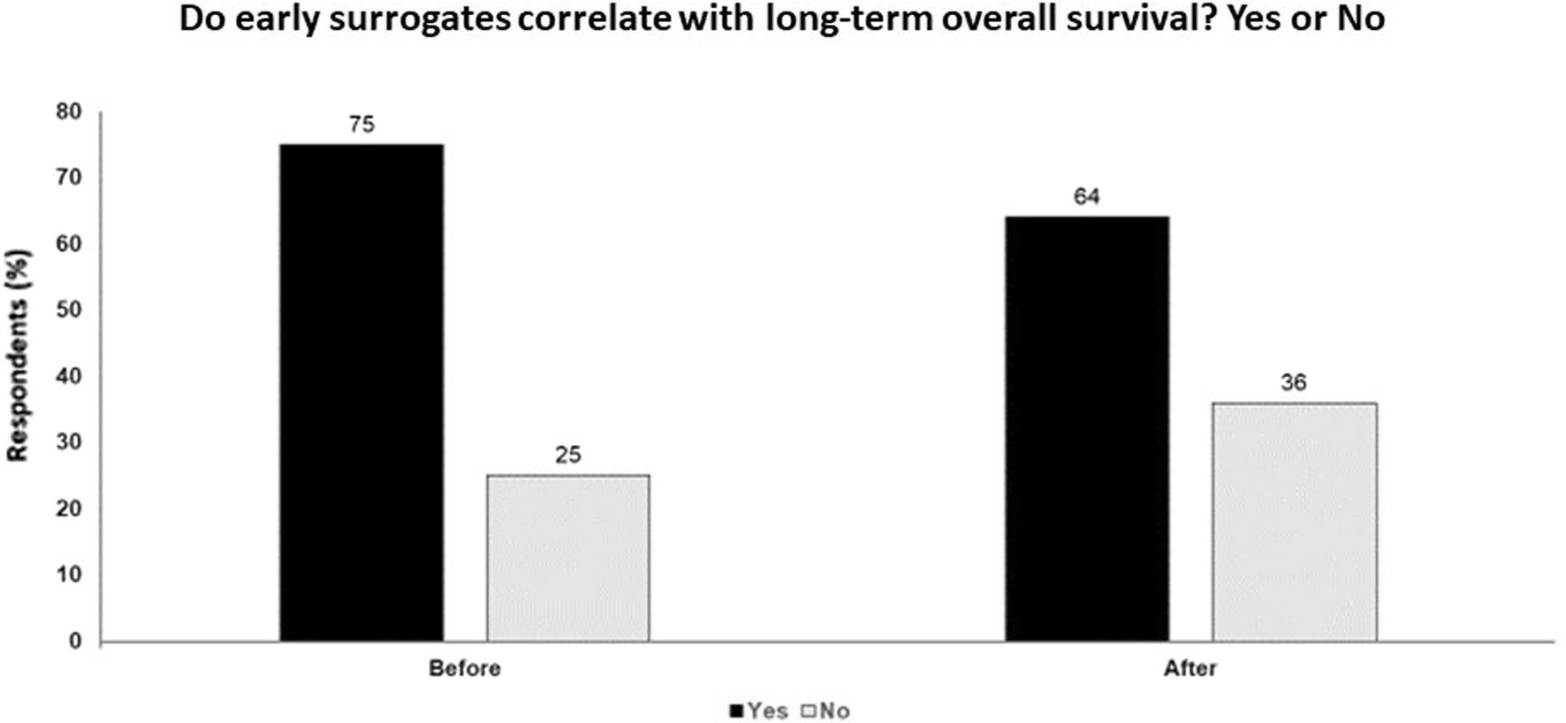
Do early surrogates correlate with long-term overall survival? Yes or no; audience response before and after debate

In conclusion, there are an increasing number of approaches to predict and better understand clinical responses to immune checkpoint inhibitors, including TMB, clonality of tumor mutations, mutations of cancer-related genes, inflammatory gene expression profiles, T cell clonality and expansion in tumor and peripheral blood, and peripheral blood monocyte subpopulations. There is now a need to integrate data into models to optimize the predictive value. Biomarkers to date cannot define which regimen to apply in which clinical setting and other clinical factors still outweigh biomarkers in treatment. The critical questions about treatment are not yet answered by biomarkers. However, I agree with Dr. Postow that early objective responses, the depth of the clinical response, and the duration of the progression-free duration do correlate with OS, but do not allow you to improve the benefit of therapy. The fate in many ways has been caste, not allowing one to better select patients for which therapy (Fig. 2).

### Key points

- Response rates in patients with melanoma treated with anti-PD-1, combined anti-PD-1 and CTLA-4, and targeted BRAF and MEK inhibition have been shown to correlate with OS.
- PFS is also predictive of OS with immune checkpoint blockade.
- Although early clinical surrogates (response rate, PFS) correlate with OS, early biological surrogates, e.g., TMB, mutations of cancer-related genes, inflammatory gene expression profiles etc., do not.
- There is a need to integrate data into models to optimize the predictive value of these biomarkers.
- To date, biomarkers cannot define which regimen to apply in which clinical setting and other clinical factors still outweigh biomarkers in treatment.

## Can vaccines transform cold into hot tumors?

### Lisa H. Butterfield: yes

Cancer vaccination is the optimal approach to promote an antitumor immune response. Tumor cells are poor antigen-presenting cells while vaccines are the key to the initiation of the tumor-immunity cycle. Why are cancer vaccines not yet more effective at driving tumor-specific immune cells into tumors to eliminate them? The one approved cancer vaccine is sipuleucel-T, an autologous cellular immunotherapy, for the treatment of patients with asymptomatic or minimally symptomatic metastatic castration-resistant prostate cancer. Sipuleucel-T promotes the recruitment of activated effector T cells into the prostate TME, indicating transformation from a cold to hot tumor [28]. Despite this, the best cancer vaccines we have are only leading to 5–10% objective tumor regressions (at best, in melanoma). One reason may be that cancer immuno-editing involves three stages: elimination, equilibrium and escape. Natural immune responses may have already eliminated the ‘easy’ tumor cell targets leaving immuno-edited and very antigenically challenging tumor cells in a challenging TME. Endogenous T cells may also be exhausted from chronic antigen stimulation from TME expression.

What vaccines can do is initiate de novo responses to new tumor-specific antigens, boost existing tumor-specific T-cell responses, and, importantly, increase epitope spreading and diversity of tumor-specific T-cell responses.

There are many components of a cancer vaccine and one approach is the dendritic cell (DC) vaccine. One complexity is that we do not know how to deliver the antigen to the DC, how to deliver the vaccine, nor how to combine it most effectively. In patients with melanoma, a DC vaccine-induced CD8+ T cell functional response in blood was associated with improved survival, and delivery before checkpoint blockade increased circulating T cell frequencies [29]. Patient-derived DCs have been shown to have reduced expression of cell surface inducible T-cell costimulator ligand (ICOSL) and defective intrinsic NF-κB signaling, which reduced priming of antigen-specific CD8+ and CD4+ T cells [30]. Increased surface ICOSL and higher extracellular soluble ICOSL also positively correlated with patient survival.

New formulations may provide greater success. FixVac (BNT111) is an intravenous liposomal RNA vaccine targeting four non-mutated, tumor-associated antigens frequently present in melanoma (NY-ESO-1, tyrosinase, MAGE-A3, and TPTE). Treatment with FixVac, as monotherapy or combined with an PD-1 checkpoint inhibitor, resulted in durable responses in patients with unresectable melanoma previously treated with a checkpoint inhibitor (vaccine alone: 3 partial responses and 7 with stable disease out of 25 patients; vaccine plus anti-PD-1: six partial responses out of 17 patients) [31]. Patients who responded also had strong CD4+ and CD8+ T cell immunity against the vaccine antigens. Vaccine-induced T cell infiltration and neo-epitope-specific killing of autologous tumour cells were observed in resected metastases [32].

Increased neoantigen load is associated with improved patient outcomes, with neoantigens targets of tumour-directed T cell responses. In melanoma, the use of neoantigen-based vaccines, with DCs loaded with short peptides, long peptides, or RNA, has been shown to be safe and feasible. Selection of epitopes that can be presented in vivo by tumour or antigen-presenting cells is an essential aspect of vaccine-targeted neoantigens. Effective neoantigen delivery, achieved through choice of formulation, immune adjuvant, delivery vehicle and dosing, will be critical for clinical utility, in combination with complementary therapies. As an example, intratumoral T cell responses were achieved in patients with glioblastoma, a tumor type which generally has a low mutational burden and is considered immunologically cold, using a neoantigen-targeting synthetic long peptide vaccine resulted [33]. Although a small study, this does provide a proof-of-principle.

### Olivera J. Finn: maybe

Therapeutic vaccination as monotherapy should no longer be considered an option since, despite many studies having been conducted with different antigens and delivery systems across various cancers, results have been disappointing. A meta-analysis of cancer vaccine trials conducted from 1999 to 2014 showed that most were phase I/II with very few having data to support progression to phase 3 trials [34]. In addition, more recent phase III vaccine trials have reported immune responses being induced to some degree but without any significant improvement in PFS or OS [35–37]. Therapeutic vaccination in combination with other immunotherapies may have some potential efficacy, although financial toxicity and lack of broad applicability, present continuous challenges.

Hot tumors are infiltrated by immune cells, T lymphocytes being especially important for antitumor effects. However, in some tumors that are considered hot based on the presence of T cells and other immune cells, the infiltrate may be primarily in the surrounding stroma and excluded from the center of the tumor, or the T cell function may be suppressed, indicating that the location and the functional state of the infiltrate is important. Therefore, some hot tumors may not really be ‘hot.’

Premalignant lesions (e.g., advanced colonic polyps) show heavy immune infiltration but the composition of the infiltrates vary considerably, from a preponderance of CD4 and CD8 T cells to mostly B cells, or primarily regulatory T cells and myeloid-derived suppressor cells. One can conclude that all premalignant polyps are hot but the assumption would have to be made, based on the examples of advanced tumors, that polyps infiltrated by regulatory T cells might most likely to progress to colon cancer. The vaccine at this stage of disease would have to change the nature of the infiltrate rather than convert cold lesions into hot. Some of these ideas were considered in a trial of therapeutic human papillomavirus-16 vaccination for vulvar high-grade squamous intraepithelial lesions, which were either very low in infiltrating immune cells (cold) or infiltrated to various degrees. High levels of infiltrating CD4 and CD8 T cells were associated with complete response after vaccination [38]. The vaccine did not increase immune infiltration of cold lesions and was not effective in their clearance. Thus, the vaccine did not change the cold microenvironment of vulvar premalignant lesions into hot but rather the hot or cold microenvironment changed, i.e. determined, the response to the vaccine.

**Fig. 3:**
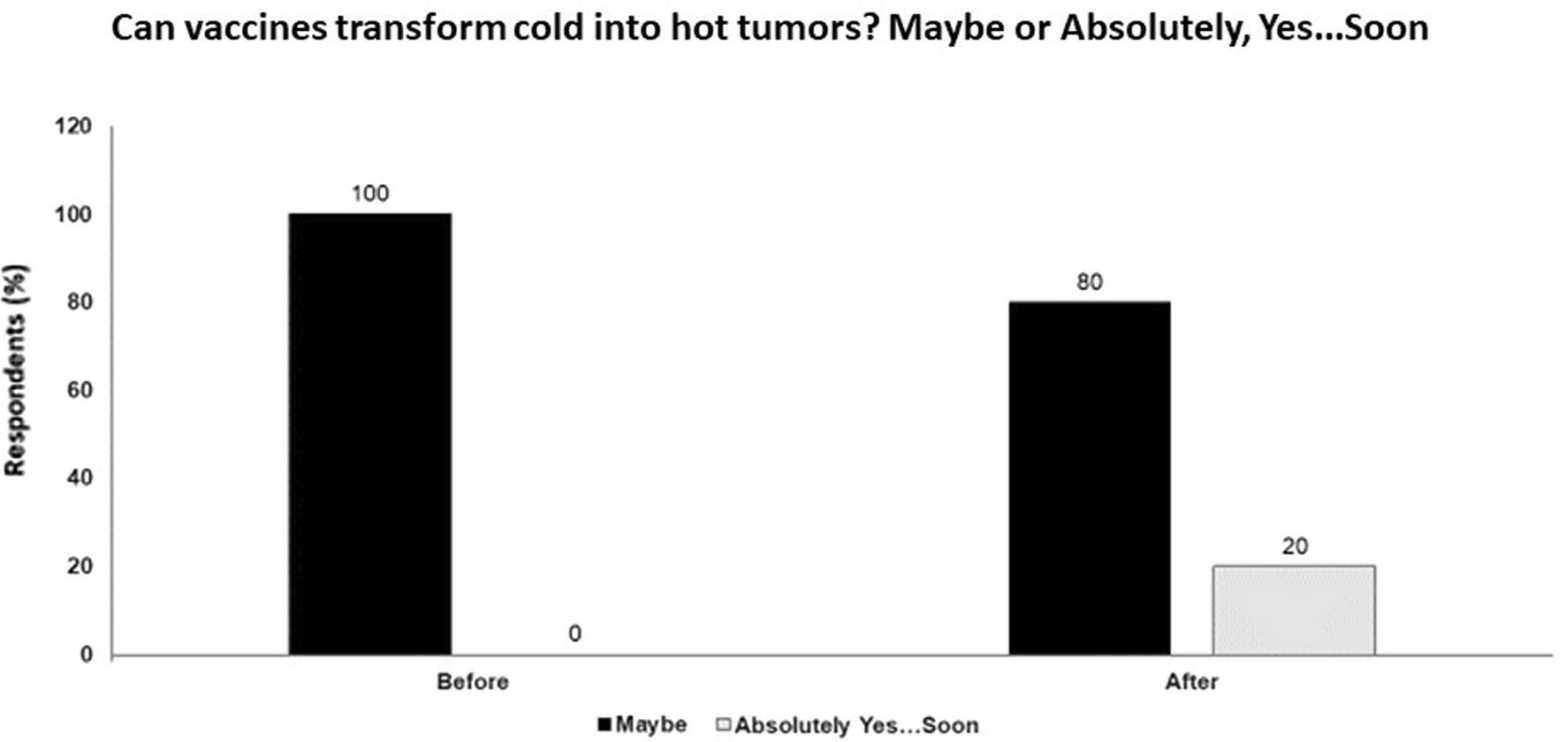
Can vaccines transform cold into hot tumors? Maybe or absolutely, yes…soon; audience response before and after debate

However, there may be some situations when vaccines might help turning cold tumors into hot tumors, namely when they are targeted against a particular family of antigens, disease-associated antigens (DAA) expressed on tumors as tumor-associated antigens (TAA). Acute inflammatory viral or bacterial infections cause changes in expression of many self-molecules giving rise to DAAs that prime specific immune memory and in patients with cancer are detected as tumor-associated antigen specific antibodies and T cells (hot patients/tumors). If these DAA/TAA-specific memory T cells are numerous and are reactivated during tumorigenesis, they may infiltrate tumors and premalignant lesions, be resistant to suppression and make those lesions ‘hot’. However, if a patient has none or very few such DAA/TAA-specific memory T cells, they may not be activated during tumor growth to expand and infiltrate the tumor and those tumors or premalignant lesions would remain cold. In this situation, vaccines against DAA/TAA may be successful in reactivating these pre-existing cells, boosting their numbers in the periphery and increasing their migration to the tumor site. Therefore, whether a vaccine could turn a cold tumor into a hot tumor may strongly depend on the vaccine antigen (Fig. 3).

### Key points

- Cancer vaccines can induce systemic T cell responses, cold tumour infiltration and objective regressions of tumors in a small minority of late-stage cancer patients with DC, other antigen-presenting cells, or mRNA and long peptides.
- Greater success is likely to be achieved with improved formulations of multiple antigen vaccines and rational combinations, including checkpoint blockade.
- Further development in oncolytic viruses, which also release and present tumor-associated antigens through tumour lysis, may also become important.
- Pre-existing immune infiltrate determines the outcome of vaccination.
- The composition and the functional state rather than the intensity of immune infiltrate determines the outcome of vaccination.
- Vaccines might be able to turn cold tumors into hot if the right antigen is chosen.

## Data science approaches to gain insights into immune-oncology development: broad versus deep analytics

### Theresa La Vallee: in favor of broad analytics

Genomics informs cancer treatment and has been most important when we have a clear driver and a drug**.** However, while immunotherapy has resulted in more usage of biomarkers, genomics has not really been able to inform decision-making. While TMB and microsatellite instability-high have provided tumor-agnostic information, genomics has not really identified significant segments of the patient population. With immunotherapy, we are considering the whole immune system, not just T cells and not just the tumor, which is obviously complex. The issue is to how to develop a treatment algorithm for each individual patient based on this complex multicomponent system. Immunotherapy response and resistance has so many mechanisms. Many of these are genetic and based in the tumor, such as the effect of oncogenes and oncoproteins, genetic and epigenetic dysfunction, or a lack of tumor antigens (i.e., low mutational burden), but there are many other factors that are also important, including TME immune suppression, inept host immunity and microbiome influence. This necessitates the need for broad immunoprofiling.

For deep tumor profiling, there are many challenges in using tumor tissue in metastatic disease. While we can learn from tumor profiling, we must remember that we are looking at a small sample from a single lesion, so this is just a snippet from the whole person. Issues with tumor tissue can be its heterogeneity, accessibility, and quality as well as preanalytical variables.

The approach successfully employed at the Parker Institute for Cancer Immunotherapy is to use a translational suite with broad longitudinal analysis of samples from tumor, blood and stool with multi-omic, multiparameter analysis. The challenge is to integrate molecular data from exome sequencing, gene expression profiling, flow cytometry, pathology imaging, cell-free DNA and proteomics, with clinical data such as treatment information, tumor measurements and RECIST reads in a useful, interpretable manner. This requires a multidisciplinary approach to derive biologically meaningful information on pharmacodynamic effects, mechanistic insights and potential biomarker candidates. Broad exploration requires collaboration and effort involving a collaborative cross-functional team with a depth of expertise (translational medicine, clinical, research, bioinformatics, biostatistics) using a science-driven approach designed to answer a clinical hypothesis and learn why it did or did not work using clinical and nonclinical data, as well as adequate time. A broad approach is needed to reveal the complexity of antitumor immunity.

### Andy Futreal: in favor of deep analytics

Deep analytics requires smaller number of patients, as opposed to a larger (broader) number. Data quality is an issue. There are many orthogonal measurements and these are too many to apply to large numbers of patients. Also, it is clear there are no simple answers or single biomarker regarding whether patients are responders or non-responders and the extent to which toxicity occurs. The full elucidation of the problem and thus its solutions will rely on integration of multiple molecular and lifestyle data measurements. However, these approaches are generally impractical and financially unfeasible to currently do with large (i.e., broad) patient numbers in the standard care setting. We need to navigate the forest to get to the other side and reduce to practice.

There are risks with using deep analytics on small numbers of patients, with bias and chance being particular issues. We also do not sample the more, let alone the most, representative patient populations. There is a real risk of overfitting in any machine learning scenario, as well as the challenge of validation in a larger patient cohort.

Our experience with one individual helps to illustrate the potential utility of deep analytics. A 77-year-old female was diagnosed with de novo stage IV M1b melanoma of unknown primary origin with left lung metastases. After wedge resection with curative intent of the solitary NRAS^Q61R^ mutated lung metastasis, her clinical course was remarkable with prolonged long-term survival which was achieved despite multiple lines of therapy for widely distributed soft tissue metastases with limited or no objective responses over an 8-year period. A whole tumor mass underwent multi-dimensional spatial and immunogenomic profiling by serial sectioning and use of alternate tumor sections for region matched IHC analyses and genomic and proteomic analyses. Individual sections were further sub-divided into up to 20 regions, producing 67 regions assessed by at least one analytical platform.

Somatic mutations in putative melanoma driver genes, including critical components of the mitogen-activated protein kinase pathway (NRAS^Q61R^, BRAF^G421R^ and MAP2K1^P124S^), were detected in all 41 regions. This supports the concept that somatic mutational heterogeneity is predominantly due to passenger mutations. A JAK1^P1044S^ mutation affecting the JAK1 activation loop was also detected in all 41 regions; this mutation resulted in signaling hypomorphism by Ba/F3 mutant transformation assay and may have contributed to the resistance to immunotherapy shown by this tumor. Diverse immune cell populations and highly localized immune activation or suppression were implicated by immune-driven transcriptional heterogeneity. Moreover, sites of similar immune composition may be spatially independent with similar immunophenotypes unrestricted by location at core or margin sites or by spatial proximity. Analysis of copy number alterations revealed that genomic intratumoral heterogeneity was driven through spatially distinct macroscopic level copy number alterations. To further explore the link between genomic copy number alterations and immune intratumoral heterogeneity, the observation of decreased estimate immune scores in regions with subclonal gain of chromosome was further investigated. Chromosome 7 gain was associated with an increase in neutrophil net activation signature and differential expression analysis comparing chromosome 7 gain versus non-gain samples showed enrichment for neutrophil-related genes and associated pathway level enrichment. This pro-neutrophil signal enrichment was associated with resistance to immune checkpoint blockade in three publicly available cohorts of immunotherapy-treated melanoma. From all of this work emerges a new piece of biological information. Although only one patient, this finding drives into larger studies and this approach from deep to broad seems to be the best way forward and is perhaps the only practical means at present.

**Fig. 4:**
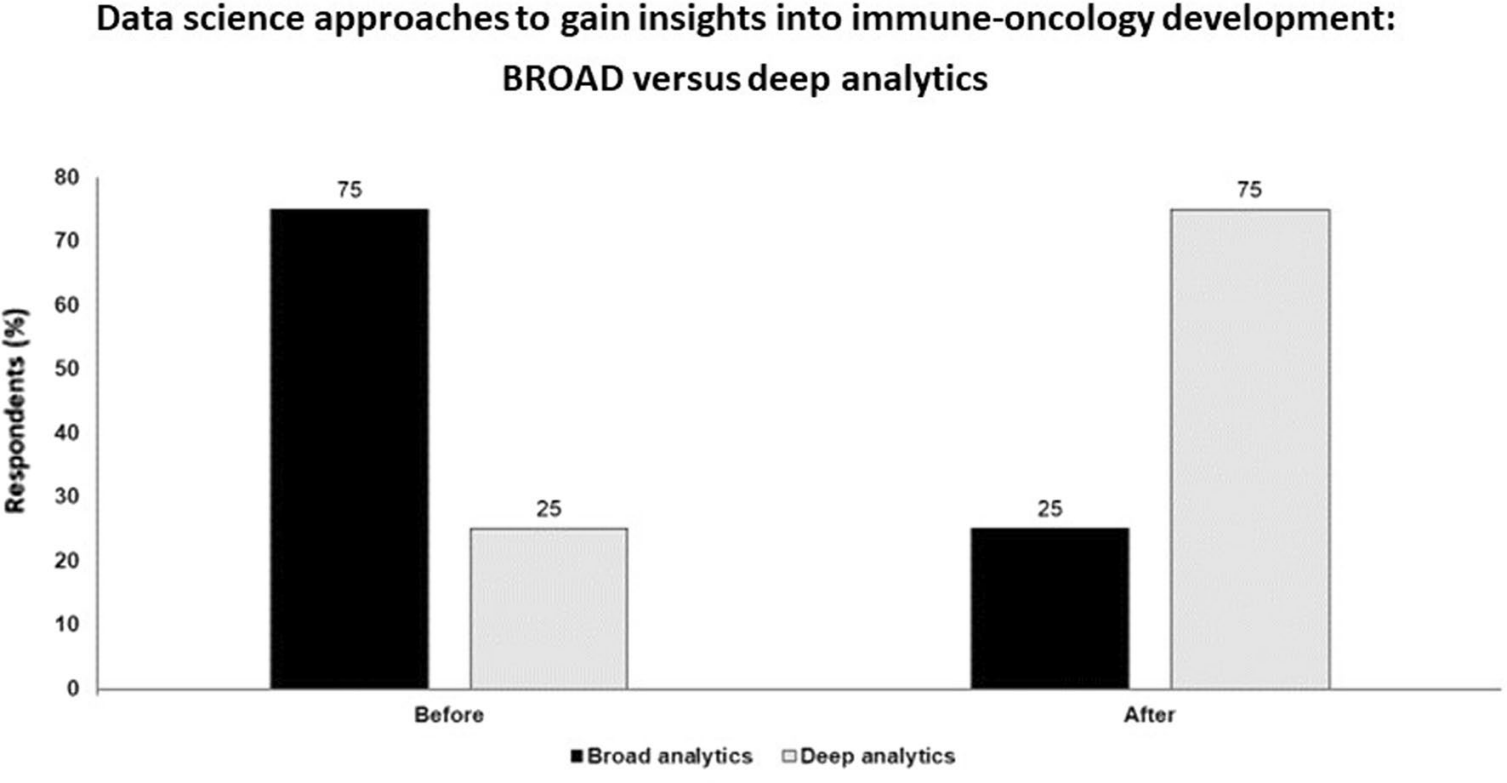
Data science approaches to gain insights into immune-oncology development: broad versus deep analytics; audience response before and after debate

There is route from deep to broad and back again. Heterogeneity is probably the biggest challenge while data may be sparse or incomplete with large numbers of patients—deep correlatives will be needed in every trial. Modelling from available data. Temporality and how the situation changes over time is an issue; better ways to sample peripheral blood and some types of tumor may address this. Finally, we need to better understand the exact nature of the antigen being presented and recognised (Fig. 4).

### Key points

- Robust predictive biomarkers for immunotherapy require evaluating the tumor and immune system and will need to use multiparameter approaches.
- Tumor tissue can be informative for biomarker evaluation but the limitations need to be appreciated including how to account for tumor heterogeneity and the multiple cell types of the immune system.

## Conclusions

There is now considerable evidence to support the use of various immunotherapeutic approaches across an increasing range of cancers. However, wider choice and greater adoption of these treatments raises new questions regarding how to optimize clinical outcomes for our patients. These Great Debate sessions provided the opportunity to weigh the evidence on five important clinical issues in immunotherapy today. Given the constraints of the format, each presentation was not intended as a rigorous assessment of the field but rather were intended to highlight some important areas of debate. We hope that these discussions can focus attention on these issues, encouraging further research on these important clinical topics.

## Abbreviations

CTLA-4: Cytotoxic T-lymphocyte-associated antigen-4
DAA: Disease-associated antigen
DAMP: Damage-associated molecular pattern
DC: Dendritic cell
FATE: Favorable antitumor effect
HR: Hazard ratio
ICOSL: Inducible T-cell costimulator ligand
IFN: Interferon
IHC: Immunohistochemistry
IRRC: Immune-related response criteria
IV: Intravenous
LAG: Lymphocyte-activation gene
NK: Natural killer
ORR: Objective response rate
OS: Overall survival
PAMP: Pathogen-associated molecular pattern
PD: Programmed death
PD-L1: Programmed death ligand
PFS: Progression-free survival
RECIST: Response Evaluation Criteria in Solid Tumors
RIG-I: Retinoic-inducible gene-I
STING: Stimulator of IFN-induced gene
TAA: Tumor-associated antigen
TIM: T cell immunoglobulin and mucin domain-containing protein
TLR: Toll‐like receptor
TMB: Tumor mutational burden
TME: Tumor microenvironment
TNBC: Triple-negative breast cancer
T-VEC: Talimogene laherparepvec
